# Possible New Focus of Diphyllobothriasis, Central Europe

**DOI:** 10.3201/eid3012.241330

**Published:** 2024-12

**Authors:** Tomáš Scholz, Roman Kuchta, Jan Brabec

**Affiliations:** Institute of Parasitology, Biology Centre of the Czech Academy of Sciences, České Budějovice, Czech Republic

**Keywords:** parasites, broad tapeworm, diphyllobothriasis, fishborne disease, Central Europe, Czech Republic

## Abstract

Diphyllobothriasis is a human parasitic infection that is widespread in the Northern Hemisphere. Popular sport fish such as pike and perch are the source of human infection. We document the autochthonous origin of diphyllobothriasis in a popular tourist destination in Central Europe, which likely marks recent colonization of the parasite.

In contrast to most human parasitic infections, which occur mainly in tropical and subtropical regions that have lower standards of hygiene and less economic development, diphyllobothriasis (also known as dibothriocephalosis) is more common in temperate and cold latitudes. The disease, which is caused by the human broad tapeworm (*Dibothriocephalus latus*), is particularly prevalent in the Northern Hemisphere. The source of infection is the consumption of popular sport fish, such as pike and perch (*1*–*4*).

There are 4 main foci of diphyllobothriasis in Europe: Fennoscandia; the Baltic region; the Alpine lakes; the Danube region; and certain areas in Russia (Figure). The number of cases decreased drastically after World War II (*4*). Today, only small foci remain, mainly in the Alpine lakes of northern Italy, Switzerland, and France, where diphyllobothriasis continues to circulate (*4*,*5*).

**Figure:**
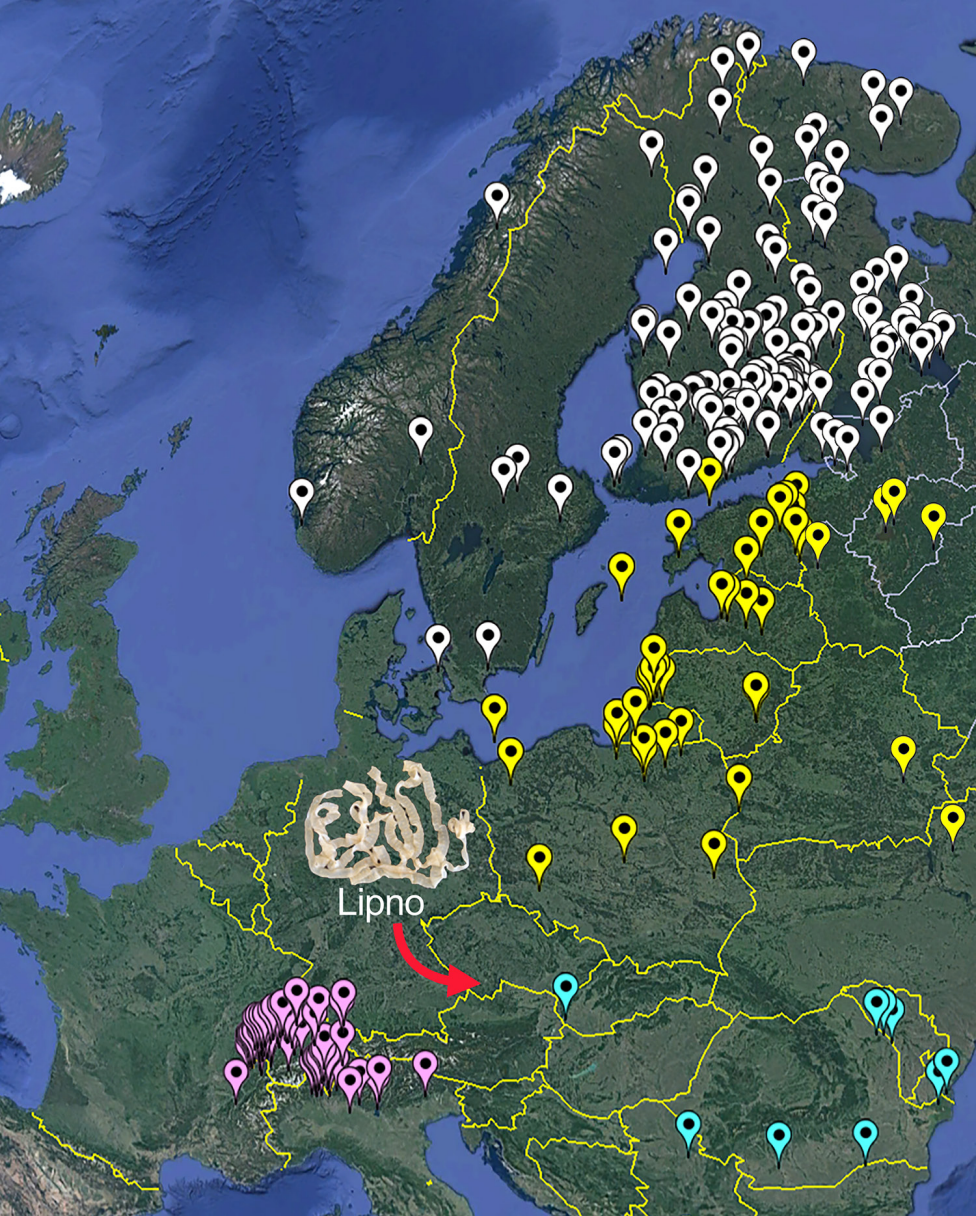
Map of the distribution of *Dibothriocephalus latus* in Europe according to Králová-Hromadová et al. (*4*) and Kuchta et al. (*8*). Diphyllobothriasis-endemic areas in Europe are identified by different colors: white indicates Fennoscandia, yellow indicates Baltic region, purple indicates Alpine lake region, and turquoise indicates Danube region. Red arrow indicates the newly reported case from the Czech Republic.

With the exception of a few imported cases, no cases of human diphyllobothriasis or of fish infected with parasite larvae have been reported in Central Europe (Czech Republic and neighboring countries such as Austria, Hungary, and eastern Germany), although the fish parasites have been intensively studied for over a century (*4*,*6*,*7*). In 2024, however, an autochthonous case of diphyllobothriasis caused by *D*. *latus* was documented in the Czech Republic.

A 37-year-old man, who had not traveled to known diphyllobothriasis-endemic areas and had not previously consumed raw or undercooked fish or fish products, obtained a pike (*Esox lucius*) caught in October 2023 near Horní Planá, the largest settlement on the Lipno reservoir (48.7 km^2^) in South Bohemia, Czech Republic (Figure). He consumed ≈1 teaspoon of raw salted roe (caviar) from the pike, which is the most common source of diphyllobothriasis in many parts of Russia (*8*). Two months later, he experienced occasional abdominal bloating. After another 2 months, he expelled a piece of tapeworm with dozens of proglottids. A coprologic examination confirmed the presence of the typical diphyllobothriid eggs. Treatment with mebendazole (Vermox, 6 tablets) (Johnson & Johnson, https://www.jnj.com) was unsuccessful, but the tapeworm was later completely expelled after a single dose of praziquantel (Biltricide) (Bayer, https://www.bayer.com). Sequencing of the complete mitochondrial cytochrome *c* oxidase subunit I gene (*cox*1) from the eggs (GenBank accession no. PQ270068) confirmed the species identity as *D*. *latus*. Because the patient could not have been infected elsewhere and his symptoms appeared after eating the pike roe, considering his infection as an autochthonous case of diphyllobothriasis is reasonable. Furthermore, raw products from fish that serve as second intermediate hosts for the human broad tapeworm (i.e., pike, perch, or ruffe) are not imported into the Czech Republic.

The Lipno Reservoir is a popular year-round tourist destination in the Czech Republic, attracting hundreds of thousands of visitors every year, including many local and foreign anglers. The fish parasites in that reservoir were intensively studied in the 1960s after the construction and filling of the reservoir (*9*). However, *D. latus* has never been found in Central Europe, apart from a single case in the 1960s in an angler from southwestern Slovakia who consumed raw perch from the Danube (*4*,*6*,*10*) (Figure). A presumably autochthonous, unpublished but molecularly identified (*cox*1; GenBank accession no. PQ270069) case from 2014 involved a 43-year-old angler from the Czech Republic who had consumed pike from northeastern and southern Bohemia, Czech Republic (as in this case) and had not traveled to diphyllobothriasis-endemic areas.

Given the intensive study of the reservoir and the popularity of the area, the parasite going undetected for decades seems unlikely. The reservoir has also never been stocked with fish imported from abroad, such as pike, perch, or ruffe, so it is unlikely that the tapeworm was imported from diphyllobothriasis-endemic areas through fish. During the examination of 108 potential second intermediate hosts (10 pike [including the pike whose eggs caused the human infection], 53 perch, and 54 ruffe) from the Lipno Reservoir in May and August 2024, no plerocercoids of *D. latus* have been found. However, the prevalence of the fish infection might be low even in other known foci of diphyllobothriasis (*5*). Therefore, a plausible explanation for the possibly autochthonous occurrence of *D*. *latus* in Central Europe is a recent appearance of the parasite in this ecosystem, probably introduced by tourists from a diphyllobothriasis-endemic area, such as the lake regions of northwestern Russia (*8*).
